# State-dependent memory and its modulation by different brain areas and neurotransmitters

**DOI:** 10.17179/excli2020-2612

**Published:** 2020-08-03

**Authors:** Mohammad-Reza Zarrindast, Fatemeh Khakpai

**Affiliations:** 1 Department of Pharmacology, School of Medicine, Tehran University of Medical Sciences, Tehran, Iran; 2 Iranian National Center for Addiction Studies, Tehran University of Medical Sciences, Tehran, Iran; 3 Cognitive and Neuroscience Research Center (CNRC), Tehran Medical Sciences, Islamic Azad University, Tehran, Iran

**Keywords:** state-dependent memory, CA1, CeA, neurotransmitter, pharmacological compounds

## Abstract

The state-dependent memory defines as a state that the retrieval of recently obtained information may be potential if the subject exists in a similar physiological situation as for the period of the encoding stage. Studies revealed that exogenous and endogenous compounds could induce state-dependent memory. The state-dependent memory made it probable to differentiate the effects of drugs per se on learning from the effects due to alterations in drug state during the task. Studies proposed the role of regions beyond the limbic formation and illustrated that state-dependent memory produced by various neurotransmitter systems and pharmacological compounds. Our review of the literature revealed that: (a) re-administration of drugs on the same state induce state-dependent memory; (b) many neurotransmitters induce endogenous state-dependent memory; (c) there are cross state-dependent learning and memory between some drugs; (d) some sites of the brain including the CA1 areas of the hippocampus, central nucleus of the amygdala (CeA), septum, ventral tegmental area (VTA), and nucleus accumbens (NAC) are involved in state-dependent memory. See also Figure 1(Fig. 1).

## Introduction

State-dependent memory is the fact that acquired information in the effect of a specific drug and/or situation may only be remembered if the organism is in a similar situation where the information was acquired (Khavandgar et al., 2002[34]). This phenomenon has been identified since 1930 and it can happen in humans by the application of numerous psychoactive compounds (Zarrindast et al., 2008[84]). There is an investigation about state-dependent memory, which reveals evidence for the effect of the state of awareness on the ability of an organism to encode memory (Girden and Culler, 1937[22]). Other investigators explored the influence of diverse situations of having the capability to learn and then remember information. Overton (1964[51]) showed that a reaction acquired in the effect of a specific drug would subsequently reoccur only when the drug situation is reinstated (Overton, 1964[51]). The concept that the organisms' situation affected their capability to recall an acquired reaction has also been indicated by other investigators. Additionally, daily life events, e.g., stress, pain, water consumption or, wake-sleep cycle influence memory processes including consolidation and reconsolidation (Nelissen et al., 2018[45]). 

When using drugs to test state-dependent learning, usually experiments are performed as follows: (a) training after saline and testing after saline administrations (S-S); (b) training after drug application followed by injecting saline (D-S); (c) training after saline administration followed injecting the drug (S-D); (d) training after drug injection, followed by testing after drug infusion (D-D). Explanation of the drug effects depends on the behavioral outcomes, as state-dependent learning develops when test performance deficiency happens in the D-S and S-D subjects, however not in the D-D subjects when compared with S-S subjects. However, state-dependent effects have shown when drugs are injected systemically, while some investigations have indicated state-dependent retention when the drugs are administered into different brain regions, suggested that state-dependent effects related to central and not peripheral effects (Jamali-Raeufy et al., 2011[32]). Also, evidence indicated that amnesic agents can produce state-dependent memory when injected during the early consolidation stage, and only if the time of the drug effect is long enough to become integrated into the memory trace. Thus, drugs' half-life and time of injection are boundary conditions creating state-dependent memory (Osorio-Gomez et al., 2019[50]). Furthermore, there are several reports that many neurotransmitter systems such as glutamatergic, GABAergic and cholinergic systems in the CA1 region of the hippocampus and central nucleus of the amygdala (CeA) may have interactions with drugs-state-dependent learning and memery (Ardjmand et al., 2011[3]; Parsaei et al., 2011[52]). Because of all that has been mentioned so far, the present review was designed to evaluate the effects of some pharmacological compounds and various neurotransmitter mechanisms as well as different brain areas in the modulation of state-dependent memory. Also, this review aimed to determine whether administration of each drug-impaired memory could also improve memory impairment if administrated again on the same state (producing state-dependent memory).

## Molecular Mechanisms of State-Dependent Memory

In normal awake situations, memory mainly depends on the excitatory transmission, particularly N-methyl-D-aspartate receptors (NMDARs), a-amino-3-hydroxy-5-methyl-4-isoxazole propionic acid receptors (AMPARs) and acetylcholine (Ach), whose function slightly predominates in the general excitatory/inhibitory balance. Nonetheless, variations of this balance in each direction can potentiate state-dependent memory. For instance, cholinergic signalings of state-dependent memory involved both inhibiting cholinergic activity by scopolamine and growing cholinergic activity by physostigmine (Jafari-Sabet et al., 2018[31]). Additionally, opiates support state-dependent memory and of all types of opioid receptors, morphine stimulated µ-receptors to appear to be the maximum effective (Zarrindast et al., 2006[87]). Notwithstanding the overhead, most of the study about state-dependent memory obtained from the stimulation of the GABAergic system and shifting excitatory/inhibitory balance toward inhibition condition (Jafari-Sabet et al., 2014[30]). A growing body of researches revealed that several neurotransmitter systems support state-dependent memory. On the other hand, various brain regions differently support state-dependent memory which will be discussed in the following sections (Radulovic et al., 2017[57]).

## Induction of Exogenous State-Dependent Memory by Systemic Injection of Addictive Drugs and Pharmacological Compounds

There is various evidence that several addictive drugs including morphine, ethanol, nicotine, amphetamine, and cocaine are involved in state-dependent learning (Rezayof et al., 2010[65]; Ardjmand et al., 2011[3]; Alijanpour and Rezayof, 2013[2]; Sanday et al., 2013[69]; Gill et al., 2015[20]). Studies revealed that verapamil, an L-type voltage-gated calcium channel blocker, and SL-327, a selective MAPK/ERK kinase inhibitor, affect morphine and ethanol state-dependent memory, as well as morphine-ethanol, morphine-nicotine, ethanol-morphine and ethanol-nicotine cross state-dependent memory (Michalak et al., 2018[37]). Moreover, some types of pharmacological compounds such as benzodiazepines, lithium chloride, caffeine, scopolamine, MK-801, and muscimol have all revealed state-dependent properties (Bouton et al., 1990[6]; Nehlig, 1999[44]; Ceretta et al., 2008[11]; Zarrindast et al., 2008[84]; Jamali-Raeufy et al., 2011[32]; Jafari-Sabet et al., 2014[30]). 

### Effect of systemic injection of morphine in the state-dependent memory

**Figure 2:**
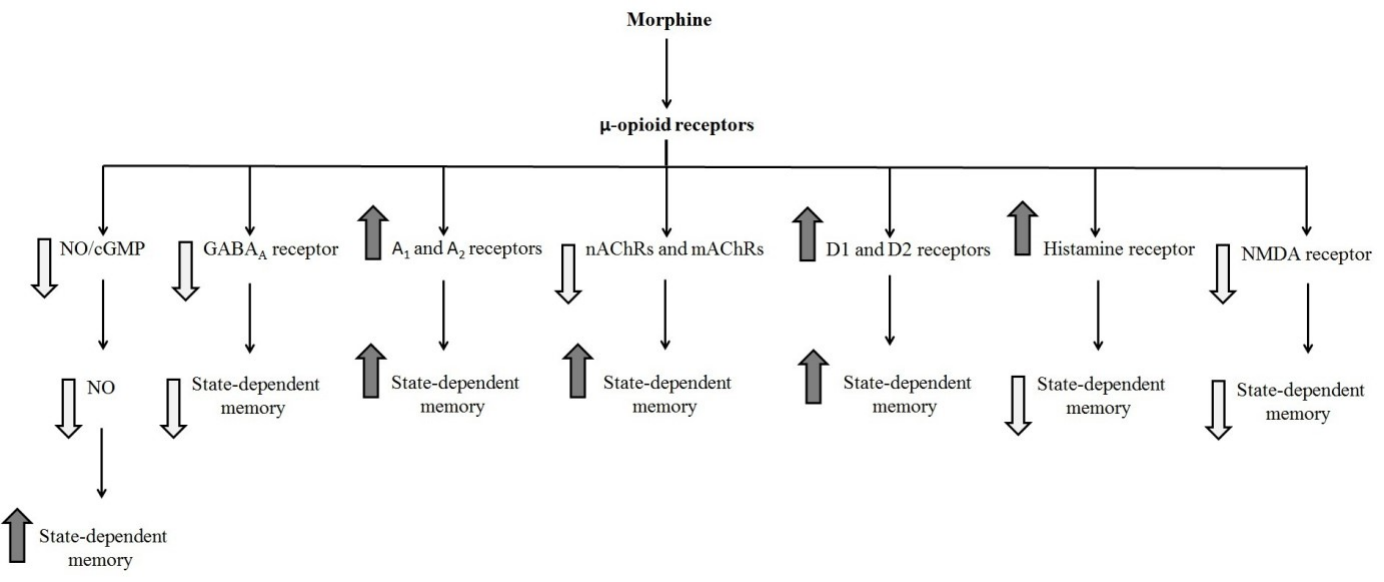
Schematic diagram of the influence of different neurotransmitters on the state-dependent memory produced by morphine. Morphine state-dependent learning could be affected by the cholinergic (Jafari et al., 2006), GABAergic (Zarrindast et al., 2006e), dopaminergic (Zarrindast et al., 2006b), histaminergic (Zarrindast et al., 2006d), glutamatergic (Zarrindast et al., 2014), and adrenergic systems (Khavandgar et al., 2002). These neurotransmitters modify morphine state-dependent memory.

Many investigations demonstrated that the opioidergic system is involved in physiological actions, e.g., anxiety, learning, and memory processes. Three different opioid receptors (i.e., µ, δ, and κ) bind to the G-protein coupled receptor superfamily and are involved in main opioid effects such as learning and memory processes. Some researchers revealed that morphine could induce dual effects on learning and memory processes (Khavandgar et al., 2002[34]). Morphine effects on the memory process depend on the time of drug injection (Nishimura et al., 1990[47]). It is well known that pre-training and post-training injection of morphine can decrease retrieval of learning tests, which is reversible via pre-test injection of morphine (Darbandi et al., 2008[13]; Ardjmand et al., 2011[3]). This event is identified as morphine state-dependent learning (Ardjmand et al., 2011[3]), which is a time- and dose-dependent process (Nishimura et al., 1990[47]). Evidence demonstrated that morphine state-dependent learning and memory produced by activation of the µ-opioid receptor, but not the κ- or δ-opioid receptors (Zarrindast et al., 2006[87]) for example, naloxone but not naltrindole antagonizes the retrieval-induced by morphine (Khavandgar et al., 2002[34]). It should be considered that naloxone at dosages of mg/kg inhibited the memory recall improvement by morphine. Nevertheless, the influences of naloxone at dosages of ng/kg were opposite to those of mg/kg dosages of the same drug, since different doses have diverse sites and mechanisms of actions (Tayebi Meybodi et al., 2005[72]). Morphine-dependent memory has been previously exhibited in y-maze discrimination, passive avoidance tasks, habituation learning, and operant tasks (Castellano, 1975[9]; Izquierdo, 1980[24]; Nishimura et al., 1990[47]; Bruins Slot and Colpaert, 1999[7]). Many neurotransmitters and drugs have been revealed to replace the pre-test administration of morphine on the restoration of memory (Vakili et al., 2004[74]). Reports are indicating that nitric oxide (NO), GABAergic, adenosinergic, cholinergic, dopaminergic, histaminergic and glutamatergic systems show a role in morphine state-dependent memory (Khavandgar et al., 2002[34], 2003[35]; Jafari et al., 2006[25]; Khalilzadeh et al., 2006[33]; Zarrindast et al., 2006[79][86];Sepehrizadeh et al., 2008[70]) (Figure 2(Fig. 2), References in Figure 2: Jafari et al., 2006[25]; Khavandgar et al., 2002[34]; Zarrindast et al., 2006[79][82][86]; Zarrindast et al., 2014[88]; Table 1(Tab. 1), References in Table 1: Ahmadi et al., 2007[1]; Azizbeigi et al., 2011[5]; Darbandi et al., 2008[13]; Khalilzadeh et al., 2006[33]; Khavandgar et al., 2002[34]; Rezayof et al., 2008[60]; Tayebi Meybodi et al., 2005[72]; Zarrindast et al., 2006[78][81][86]). 

It is noteworthy that in addition to the systemic injection of morphine, intra-cerebral injection of morphine also induces a state-dependent memory. That way, pre-test Intra-CA1 administration of morphine also inhibited amnesia produced by pre-training Intra-CA1 injection of norharmane and vice versa. Upon this result, it has been suggested that there may be a cross state-dependent memory retrieval between morphine and norharmane of which the µ-opioid receptor has a key role in this process (Ebrahimi-Ghiri et al., 2019[14]). Also, amnesia-induced by Intra-CA1 administration of tramadol improved by pre-test injection of either tramadol or morphine. It seems that tramadol is capable of produced state-dependent memory and also, it has a cross state-dependent memory with morphine in the CA1 areas of the hippocampus, done possibly via a µ-opioid receptor (Niknamfar et al., 2019[46]).

### Effect of systemic injection of alcohol in the state-dependent memory

**Figure 3:**
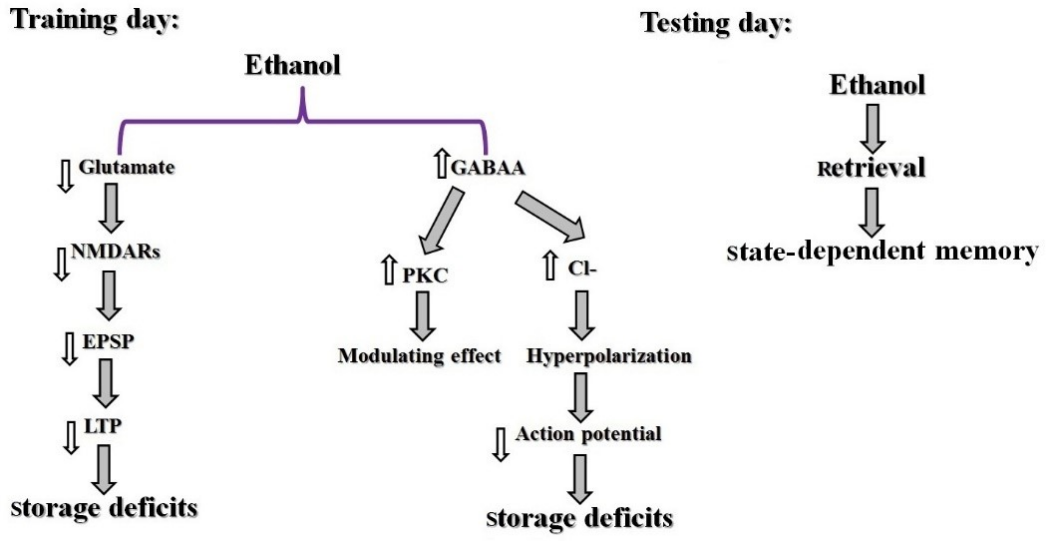
Schematic model of ethanol-induced state-dependent memory. Ethanol activates GABA_A_ receptors which conduct Cl^-^, resulting in neuronal hyperpolarization. Furthermore, PKC has a modulatory effect in the response of the GABA_A_ receptor to ethanol. Also, ethanol influences NMDA receptors which lead to inhibition of EPSP which decreased LTP. It is possible that ethanol causes storage deficiency in the training day and does not affect retrieval in the testing day hence producing a state-dependent memory (Miller et al., 1978).

Studies in laboratory animals demonstrated that abused compounds, for example, alcohol and opiates show very strong signs for producing state-dependent learning and memory. Also, some documents exhibited that ethanol could produce state-dependent memory in alcoholic people (Rezayof et al., 2010[65]). So, pre-train infusion of a higher dosage of ethanol has an impairing influence on memory in a passive avoidance test, on the other hand, pre-test injection of a lower dosage of ethanol could inverse the memory impairment (Rezayof et al., 2007[62]). According to investigations, the ethanol-produced state-dependent learning and memory is modulated by Ach, dopamine (DA), NMDA, GABA, cannabinoid, serotonin, morphine, adrenergic and NO systems (Table 2(Tab. 2); References in Table 2: Nakagawa and Iwasaki, 1996[42]; Rezayof et al., 2007[62], 2008[59][63], 2010[65]; Zarrindast et al., 2013[83]) (Nakagawa and Iwasaki, 1996[42]; Vakili et al., 2004[74]; Rezayof et al., 2007[62], 2010[64][65]; Alijanpour and Rezayof, 2013[2]; Zarrindast et al., 2013[83]). There are reports that alcohol activates GABA receptors, specifically type GABA_A_, which conduct Cl^-^ resulting in neuronal hyperpolarization. Furthermore, protein kinase C (PKC) plays a modulatory role in the response of GABA_A_ receptors to alcohol. Also, ethanol affects NMDA receptors, which cause inhibition of the excitatory postsynaptic potential (EPSP). Consequently, this inhibition leads to decreased long-term potentiation (LTP) in the hippocampal formation. Because alcohol induces storage deficiency rather than retrieval deficiency, alcohol may produce state-dependent memory (Figure 3(Fig. 3)) (Miller et al., 1978[38]). 

## Involvement of Various Brain Areas in the State-Dependent Memory

**Figure 4:**
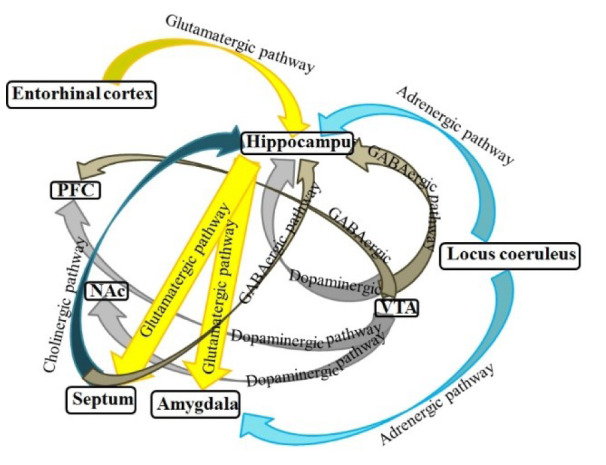
Schematic model of possible pathways for state-dependent memory in the brain. The hippocampus, septum, amygdala, VTA, NAc, and cortex form a complex network of brain systems for modulation of state-dependent memory (Jafari-Sabet and Jannat-Dastjerdi, 2009)

There is much evidence demonstrating that not only state-dependent memory is produced by the systemic administration of drugs but also is induced by the injection of drugs into the various brain areas. Several studies in our laboratory revealed that various neurotransmitter mechanisms, e.g., glutamatergic, GABAergic and cholinergic systems of the CA1 areas of the hippocampus and CeA have interaction with drug state-dependent memory (Figure 4(Fig. 4); Reference in Figure 4: Jafari-Sabet and Jannat-Dastjerdi, 2009[28]) (Ardjmand et al., 2011[3]; Parsaei et al., 2011[52]).

### Involvement of the hippocampus in the state-dependent memory

The hippocampus as a part of the limbic structure is well known as the main area of synaptic plasticity, LTP, integration of information, encoding, acquisition, consolidation, and retrieval of memory. It has been indicated that memory formation and the retrieval process may share various molecular signaling in the hippocampus and that the retrieval process starts extinction needing stimulation of some signaling cascades as well as protein synthesis (Morris, 2003[39]). Moreover, the hippocampus is a critical site for modulation of the state-dependent memory process (Rezayof et al., 2007[62], 2010[65]). The dorsal hippocampal opioidergic, dopaminergic, cholinergic, glutamatergic, cannabinoid, α2-adrenergic, NO and GABAergic systems may play a key role in mediating of state-dependent memory (Rezayof et al., 2007[62], 2008[63]; Jafari-Sabet, 2011[26]; Parsaei et al., 2011[52]; Piri and Zarrindast, 2011[54]; Jafari-Sabet et al., 2014[30]; Ebrahimi-Ghiri et al., 2019[14]).

### Involvement of the hippocampal adrenergic system in the state-dependent memory 

Monoaminergic systems are remarkably involved in many behavioral reactions, for example, learning and memory consolidation. The ascending noradrenergic system originates from the locus coeruleus and innervates the neocortex and hippocampus (Azami et al., 2010[4]). It has been demonstrated that α-adrenergic receptors of the CA1 areas of the hippocampus show main regulatory roles in the modulation of memory consolidation, and reconsolidation processes (Azami et al., 2010[4]; Piri and Zarrindast, 2011[54]). Many investigations revealed that the administration of noradrenaline into the CA1 areas of the hippocampus enhances memory formation (Piri and Zarrindast, 2011[54]). Also, reports are indicating that noradrenergic receptors of the CA1 areas of the hippocampus play a role in state-dependent learning and memory (Azami et al., 2010[4]; Piri and Zarrindast, 2011[54]). It has been demonstrated that state-dependent amnesia, produced by peripheral epinephrine, may happen physiologically and may be mediated by the brain norepinephrine acting on the nucleus tractus solitarius and hippocampus and amygdala. Norepinephrine has been revealed to be involved in state-dependent fear memories (Rosa et al., 2014[68]). 

Further supporting the participation of noradrenergic receptors mechanism in the state-dependent memory, additional experiments were carried out by the blocking of noradrenergic receptors in the CA1 areas of the hippocampus. The findings revealed that stimulation of the α- and β-adrenergic receptors in the dorsal hippocampal CA1 areas modulate drug state-dependent memory (Piri and Zarrindast, 2011[54]; Zarrindast et al., 2013[83]). One of the main physiological functions of α2-adrenoceptor activation is the modulation of the other neurotransmitters released in other regions of the brain. Some evidence displayed that α2-adrenoceptor stimulation enhanced the basal release of GABA in the hippocampus, NAC, striatum, and cortex. Studies revealed that the agonists and antagonists of α2-adrenoceptor control memory retrieval through increasing or declining basal GABA release, respectively (Pittaluga and Raiteri, 1987[55]). Moreover, investigations indicated that α2-adrenoceptors of the CA1 areas of the hippocampus play a modulatory role in muscimol state-dependent memory. α2-adrenoceptor activation in the CA1 regions of the hippocampus may be coupled to GABA release from the hippocampus. This phenomenon may regulate memory retrieval in the hippocampus (Jafari-Sabet et al., 2013[27]). Additionally, studies demonstrated that the α2-noradrenergic receptors of the CA1 regions could influence WIN55,212-2 state-dependent learning in a passive avoidance task (Piri and Zarrindast, 2011[54]). There is a report showing that the α1- and α2-noradrenergic receptors of the CA1 regions play a role in scopolamine state-dependent memory (Azami et al., 2010[4]).

Although the expression of the β-adrenergic receptor in the brain is considerably lesser than the α receptors, they are important for cognitive functions (Ghiasvand et al., 2011[17]). The β-noradrenergic receptors of the CA1 areas mediated the ethanol-induced state-dependent retrieval (Zarrindast et al., 2013[83]). Otherwise, the state-dependent memory of social recognition induced by some drugs such as methylphenidate depends on the effect of both catecholamines on the ventromedial prefrontal cortex, although norepinephrine inhibits the social recognition memory in the hippocampus (Garrido Zinn et al., 2018[15]). 

### Involvement of the hippocampal GABA-ergic system in the state-dependent memory 

The main inhibitory neurotransmitter in the central nervous system (CNS) is the γ-aminobutyric acid (GABA), which affects learning and memory processes (Parsaei et al., 2011[52]; Jafari-Sabet et al., 2014[30]). The hippocampus has numerous GABAergic interneurons and these interneurons project from the septum. The CA1 GABA receptors play a key role in learning and memory processes through the modulation of information processing in the hippocampus (Nazari-Serenjeh et al., 2011[43]). It is demonstrated that Intra-CA1 administrations of GABA_A_ receptor agonists decrease memory, whereas their antagonists increase retrieval in different tasks. Some investigations indicated that the GABA_A_ receptors of the CA1 regions of the hippocampus also participate in state-dependent memory (Parsaei et al., 2011[52]). It is thought that GABA_A_ agonists, e.g., muscimol, produce drug state by acting on the chloride ion channels (Nakagawa et al., 1995[41]). Additionally, reports are indicating that opioidergic, muscarinic cholinergic, α2-adrenergic, and NO systems of the CA1 areas of the hippocampus are important for inducing muscimol-related state-dependent memory (Jafari-Sabet and Jannat-Dastjerdi, 2009[28]; Jafari-Sabet, 2011[26]; Jafari-Sabet et al., 2013[27], 2014[30]).

Also, some documents showed the involvement of the GABAergic system of the CA1 areas of the hippocampus in the modulation of drug state-dependent memory. Pre-test injection of bicuculline in the CA1 areas of the hippocampus increased the effect of pre-test lithium on the reversal of memory, which supports the role of GABA receptors in the CA1 areas of the hippocampus in the effects of lithium on state-dependent learning (Parsaei et al., 2011[52]). Additionally, researchers also suggested that there is an interaction among the GABAergic and opioidergic systems of the CA1 areas of the hippocampus in learning and memory (Jafari-Sabet and Jannat-Dastjerdi, 2009[28]). Studies revealed that GABAergic and opioidergic mechanisms are interconnected via µ-opioid receptors. These receptors are present exclusively on the inhibitory interneurons (principally GABAergic) in the hippocampus. Stimulation of the µ-opioid receptors has been displayed to hyperpolarize interneurons and presynaptically inhibited the GABA release in the hippocampus (Cohen et al., 1992[12]). It seems that the µ-opioid receptors of the dorsal hippocampus are involved in muscimol state-dependent memory by inhibition of GABA release (Jafari-Sabet and Jannat-Dastjerdi, 2009[28]).

Some evidence showed that the α2-adrenoceptor stimulation enhanced the basal GABA release in the cerebral cortex, hippocampus, and striatum. The α2-adrenoceptors of the dorsal hippocampus play a key function in muscimol state-dependent memory (Jafari-Sabet et al., 2013[27]). Furthermore, studies revealed that there is an interaction among the GABAergic and NO systems in the experimental animals, regarding memory consolidation. There is a correlation between a reduction in the NO release in the CA1 and the physiological condition under which muscimol facilitates memory retrieval. Thus, the NO system in the dorsal hippocampus mediated the muscimol state-dependent retrieval (Jafari-Sabet et al., 2014[30]). 

### Involvement of the hippocampal glutamatergic system in the state-dependent memory 

One of the most frequent amino acids in the CNS is glutamate, which acts as an excitatory neurotransmitter. The NMDA receptors highly distribute in the brain, such as the cortex, hippocampus, septum, amygdala, and NAC. The maximum concentrations of NMDA receptors are expressed in the hippocampal CA1 regions. The main glutamatergic afferent to the hippocampus is projected through the pyramidal neurons in layers II and III of the entorhinal cortex (Zarrindast et al., 2012[85]). The NMDA receptors of the hippocampus appear to be an essential upstream stage for the initiation of intracellular cascades including gene expression, protein synthesis, cAMP/PKA/CREB signaling, and remodeling of synaptic contacts during learning (Cammarota et al., 2000[8]). Also, the hippocampal NMDA receptors show a key role in the modulation of synaptic plasticity, LTP, and short- and long-term memories (Morris, 2003[39]; Rezayof et al., 2010[64]). The hippocampal NMDA receptors have a role in state-dependent learning, too. Investigations revealed that NMDA receptor antagonists prevent the occurrence of LTP in the hippocampus and also decrease several forms of learning and conditioning, hence showing that the NMDA receptors of the hippocampus have a modulatory role in state-dependent learning. Studies indicated that NMDA receptor antagonists such as ketamine, phencyclidine, dextromethorphan, MK-801, and CGS 19755 induce state-dependent retrieval (Zarrindast et al., 2014[88]). Also, arcaine and MK-801 by decreasing the NMDA receptor function induce state-dependent recall (Ceretta et al., 2008[11]). Moreover, the hippocampal NMDA receptors display the main role in the modulation of drug state-dependent learning and memory, for instance, ethanol state-dependent learning. The NMDA receptors are molecular targets for the function of ethanol on the hippocampal glutamatergic neurons (Rezayof et al., 2008[63]). It has been shown that acute ethanol withdrawal indicates a powerful excitatory and neurodegenerative influence on the hippocampal NMDA receptors. In this respect, researches revealed that ethanol produced variations in the CA1 glutamatergic neurotransmission via the NMDA receptors. These receptors have a vital role in ethanol's influences on neural development, synaptic plasticity, learning, and memory (Prendergast et al., 2004[56]). 

### Involvement of the hippocampal cannabinoid system in the state-dependent memory 

Cannabinoids as the psychoactive agents have a vital neuromodulatory effect in some behavioral responses, e.g., learning and memory. The cannabinoid CB1 receptors are extensively distributed in the hippocampus, amygdala, cerebral cortex, and NAC. The CB1 receptors are enriched in the hippocampal formation, especially in the dorsal hippocampus. The CB1 receptors of the CA1 areas of the hippocampus have also been revealed to produce state-dependent memory, as pre- or post-train Intra-CA1 injections of WIN55,212-2, a synthetic cannabinoid CB1/CB2 receptor agonist, decreased retrieval of learned tests which was restored via pre-test Intra-CA1 infusion of the drug, indicating the drug state-dependent memory (Moshfegh et al., 2011[40]). In the hippocampal formation, cannabinoid CB1 receptors are placed in the presynaptic location of the GABAergic axon terminals. Endogenous cannabinoid mediates retrograde signaling, which may be participated in the inhibition of many neurotransmitter releases, e.g., glutamate, Ach and noradrenaline (Shen et al., 1996[71]; Gifford et al., 1997[19]). Recent investigations have revealed that endocannabinoids as the retrograde signaling molecules, i.e., being produced in a Ca^2+^-dependent process from post-synaptic terminals, then diffusing to the presynaptic terminals and so preventing neurotransmitter release. This presynaptic inhibition of neurotransmitters released by cannabis is reinforced by the fact that CB1 receptors are found at a higher density at the pre-synaptic terminal compared to the soma, and that they can intensify the inhibition of voltage-dependent Ca^2+^ channels by decreasing the duration of the action potential by activation of two different types of K^+^ channels (Wilson and Nicoll, 2002[75]). The hippocampal cannabinoid system may interact with other neurotransmitter mechanisms for modulation of state-dependent memory. Some evidence showed that there is an interaction between the CA1 cannabinoid and DA, GABA as well as Ach systems in the modulation of state-dependent memory (Zarrindast et al., 2010[80]; Jamali-Raeufy et al., 2011[32]; Jafari-Sabet and Karimi, 2017[29]). 

### Involvement of the hippocampal cholinergic system in the state-dependent memory 

Ach is a well-known neurotransmitter in the CNS which is involved in learning, memory, and attention processes. The hippocampus is cholinergically innervated by the medial septum area and the vertical limb of the diagonal band of Broca. In the hippocampus, both nAChRs and mAChRs are distributed at the presynaptic and postsynaptic positions of both principal neurons and inhibitory interneurons, where they produce potent bi-directional effects on the synaptic transmission. Furthermore, there is the interplay between the cholinergic system and extracellular signal-regulated kinase as well as the mammalian target of rapamycin pathways in the improvement of short- and long-term memories for the duration of the acquisition and recall of the passive avoidance in the hippocampus (Giovannini et al., 2015[21]). Moreover, the hippocampal cholinergic system (both nAChRs and mAChRs) participated in the state-dependent retrieval of memory (Jamali-Raeufy et al., 2011[32]). Several investigations reported that the nAChRs are involved in morphine, ethanol, muscimol, WIN55,212-2, α1- and α2-adrenergic receptors as well as DA D1 receptors state-dependent learning and memory (Rezvani and Levin, 2003[67]; Rezayof et al., 2008[60]; Azami et al., 2010[4]; Jafari-Sabet, 2011[26]; Alijanpour and Rezayof, 2013[2]; Piri et al., 2013[53]). Also, some studies in our laboratory revealed that the nAChRs of the CA1 areas of the hippocampus play the main role in cross-state dependencies such as ethanol, and WIN55,212-2 (Table 3(Tab. 3); References in Table 3: Azami et al., 2010[4]; Jafari-Sabet, 2011[26]; Jamali-Raeufy et al., 2011[32]; Piri et al., 2013[53]; Rezayof et al., 2008[59]) (Rezayof et al., 2008[59]; Jamali-Raeufy et al., 2011[32]; Alijanpour and Rezayof, 2013[2]). The participation of mAChRs of the CA1 areas of the hippocampus in state-dependent learning and memory revealed by experimental investigations showed that amnesia produced via post-train Intra-CA1 microinjection of scopolamine was completely restored through pre-test Intra-CA1 injection of the same dose of scopolamine (Azami et al., 2010[4]; Jamali-Raeufy et al., 2011[32]). Since scopolamine predominantly impairs acquisition processes and can even facilitate consolidation, thus scopolamine could induce state-dependent memory (Klinkenberg and Blokland, 2010[36]). Also, the CA1 mAChRs is involved in modulation of drug state-dependent learning and memory including WIN55,212-2, ethanol, muscimol, and DA D1 receptor agents (Rezayof et al., 2008[59]; Jafari-Sabet, 2011[26]; Jamali-Raeufy et al., 2011[32]; Piri et al., 2013[53]). 

### Involvement of the hippocampal dopaminergic system in the state-dependent memory 

One of the most important catecholamine neurotransmitters in the brain is DA (Nazari-Serenjeh et al., 2011[43]). The hippocampus receives the dopaminergic input from the mesolimbic structures, e.g., the VTA and substantia nigra pars compacta (SNc) (Zarrindast et al., 2010[80]). It has been revealed that DA D1- and D2-like receptor-expressing neurons are severely distributed in the rodent's hippocampus. Activation of the dopaminergic receptors is essential for the consolidating of LTP, learning, and memory in the CA1 regions. Studies also exhibited that the hippocampal dopaminergic system is involved in state-dependent memory (Rezayof et al., 2007[62]; Zarrindast et al., 2010[80]; Piri et al., 2013[53]).

Some evidence reported that DA D1/D5 receptors are participating in a proliferation of state-dependent memory not only in the hippocampal formation but, remarkably, in regions of the brain associated with an acquaintance. Furthermore, our previous investigations also proposed that the hippocampal DA D1-like and D2-like receptors have a key function in drug state-dependent learning, for instance, ethanol, scopolamine, and lithium (Rezayof et al., 2007[62]; Zarrindast et al., 2009[76]; Zarrindast et al., 2010[80]; Piri et al., 2013[53]). 

In conclusion, the hippocampus due to its important role in the modulation of different models of memories and including different neurotransmitter systems plays a key role in the modulation of state-dependent memory. As mentioned above, although Intra-CA1 administration of different drugs impaired memory, Intra-CA1 re-administration of the drugs on the same state could improve memory (producing state-dependent memory). This confirms the well-known role of the hippocampus in the modulation of memory processes.

### Involvement of the amygdala in the state-dependent memory

The amygdala is a prominent limbic formation that comprises four nuclear regions. The CeA is the key output of the amygdala, which is exceedingly participating in emotional learning, reward-related learning, and memory retrieval (Ardjmand et al., 2011[3]). Also, the CeA is involved in state-dependent memory, as it displays a key function in state-dependent memory produced by opioid, cannabinoid, GABA, glutamate, Ach and β1-noradrenergic mechanisms (Rezayof et al., 2009[61]; Rassouli et al., 2010[58]; Ardjmand et al., 2011[3]; Ghiasvand et al., 2011[17]). 

### Involvement of the amygdala cholinergic system in the state-dependent memory 

The amygdala received cholinergic projections from the nucleus basalis magn-ocellularis (NBM). Immunocytochemical studies showed that the mAChR is found in the amygdala, hippocampus, and neocortex, which are participating in cognitive actions for instance learning and memory processes. In the CeA a large difference in the mAChR immunoreactivity was detected, regarding the number of neurons as well as their staining intensity. Studies exhibited that there is a maximum number of mAChRs in the CeA, which show a critical function in the production of LTP in the amygdala. Some documents showed the participation of the amygdala cholinergic mechanism in memory storage. It has been indicated that the administration of cholinergic receptor antagonists within the amygdala decline learning and memory processes in diverse behavioral tests. As well, the CeA mAChR plays a key role in state-dependent memory. There is a probability that the mAChR produced state-dependent memory retrieval is conducted through interaction with other mechanisms in the amygdala. The mAChRs of the CeA play the main role in morphine-produced state-dependent memory in the inhibitory avoidance learning test (Rezayof et al., 2009[61]). Moreover, the cholinergic and serotonergic receptors of the CeA have a functional interaction with morphine-produced state-dependent memory in the inhibitory avoidance learning test. The rewarding effects of the drugs likely lead to produce cross state-dependent memory among the drugs (Tirgar et al., 2014[73]).

### Involvement of the amygdala glutamatergic system in the state-dependent memory 

Studies demonstrated that the NMDA receptors are expressed in widespread regions of the CNS, including the limbic regions and especially the CeA. The CeA receives sensory information through the glutamatergic afferents from the thalamus, prefrontal cortex, and hippocampal formation as well as basolateral amygdala (BLA). Ultra-structural studies have displayed that the NMDA receptors are distributed in the dendrites of the CeA neurons and are participating in the synaptic plasticity, LTP, and emotional learning and memory in the CeA. Many experiments demonstrated that the administration of both competitive and non-competitive NMDA receptor antagonists into the amygdala impaired learning and memory. Also, the CeA glutamatergic neurons through the NMDA receptors play a key role in state-dependent memory. State-dependent memory related to the NMDA receptors of the CeA may be due to reward-related learning. Furthermore, it has been reported that pre-test Intra-CeA infusion of NMDA along with morphine reversed memory impairment induced by post-train injection of morphine, suggesting that the NMDA receptors of the CeA participate in morphine state-dependent memory (Ardjmand et al., 2011[3]). This phenomenon is due to the role of drugs and the CeA in reward-related learning. Also, the NMDA receptors of the CeA may show a function in cannabinoid state-dependent memory. For instance, reduction of consolidation produced by Intra-CeA injection of ACPA is performed, somewhat, via an NMDA receptor mechanism in the CeA (Ghiasvand et al., 2011[18]). 

### Involvement of the amygdala cannabinoid system in the state-dependent memory 

In the CeA, cannabinoid CB1 receptors were situated on the GABAergic intercalated cells and the glutamatergic neurons, which may prevent both GABA and glutamate release. Behavioral studies exhibited that post-train Intra-CeA injections of WIN55,212-2 produced inhibitory avoidance memory deficiency while pre-test Intra-CeA injections of a similar dosage of WIN55,212-2 reversed the impairment of inhibitory avoidance memory retrieval that was indicative of drug-related state-dependent memory (Ghiasvand et al., 2011[17]; Piri and Zarrindast, 2011[54]). The consolidation and retrieval of inhibitory avoidance memory has revealed the need for intact glutamate receptors. Probably, a state-dependent memory that is produced via Intra-CeA injection of WIN55,212-2 may affect the prevention of glutamate release in the CeA (Haller et al., 2007[23]). State-dependent memory which is produced via WIN55,212-2 seems to be dependent on the rewarding properties of the cannabinoids. Regarding the latter proposition, it has been indicated that WIN55,212-2 increased the neuronal firing of the dopaminergic neurons in the forebrain reward sites (Gessa et al., 1998[16]).

Interestingly, there is cross state-dependent memory retrieval between morphine and ethanol that the BLA CB1 cannabinoid receptors are involved in this process. The rewarding effects of the drugs may participate in this phenomenon (Ofogh et al., 2016[49]). 

### Involvement of the amygdala GABAergic system in the state-dependent memory 

Investigations demonstrated that the CeA neurons and their efferent projections are mainly the GABAergic neurons. The CeA neurons receive the GABAergic afferents from the intercalated neurons. Before, the participation of the CeA GABAergic mechanism on the learning and memory retention had been reported. Intra-amygdala injection of the GABAergic receptor agonists decreased memory, whereas their antagonists increased memory storage and retrieval in the inhibitory avoidance tests (Castellano et al., 1989[10]). Some investigation indicated that the CeA GABA_A_ receptors also participated in the state-dependent memory process using the passive avoidance task (Rassouli et al., 2010[58]). As the CeA and GABAergic mechanisms participate in reward-associated learning, the importance of the amygdala GABAergic mechanism in the state-dependent memory seems likely (Rezayof et al., 2002[66]; Zarrindast et al., 2004[77]; Zhu and Pan, 2004[90]). So, pre-test Intra-CeA injection of muscimol impaired morphine state-dependent memory, suggesting the role of CeA GABA_A_ receptors in morphine state-dependent memory (Rassouli et al., 2010[58]).

### Involvement of the amygdala adrenergic system in the state-dependent memory

The CeA receives vast noradrenergic projections from the locus coeruleus. Consistent with investigations, the amygdala adrenergic receptors modulated inhibitory avoidance memory. β-adrenoceptors of the amygdala participated in the regulation of retrieval of one-trial step-down inhibitory avoidance test. Growing documents displayed that intra-amygdala injections of β-adrenoreceptor agonists improved the retention process in either spatial or emotionally-motivated memory tests, but their antagonists decreased learning and memory in the behavioral studies. Some evidence revealed the participation of the CeA noradrenergic receptors in state-dependent memory (Ghiasvand et al., 2011[17]). Moreover, there is evidence showing an interplay among the β1-noradrenergic and cannabinoid systems of the CeA on the retrieval stage of state-dependent memory produced via WIN55,212-2. Hence, it can be said that noradrenergic and cannabinoid mechanisms in the CeA may regulate memory retrieval (Ghiasvand et al., 2011[17]; Moshfegh et al., 2011[40]). 

### Involvement of the amygdala opioidergic system in the state-dependent memory 

Immunocytochemical researches have revealed that the opioid receptors, particularly µ-opioid receptors, are present in the dendrites of the CeA. It has been reported that the opioidergic system of the CeA involved in the neural signaling, memory, reward behaviors, and plasticity are related to addictive behaviors (Zhu and Pan, 2004[90]). There is also evidence that the CeA opioidergic mechanism has a function in state-dependent learning (Rezayof et al., 2009[61]; Rassouli et al., 2010[58]; Ardjmand et al., 2011[3]). Morphine state-dependent learning is correlated with the reward properties of morphine and the CeA plays a key action in the acquisition and expression of the morphine reward effects (Rezayof et al., 2002[66]). 

Since the amygdala has several neurotransmitter systems as well as various afferent and efferent projections different processes of memory, such as state-dependent memory, could be modulated.

### Involvement of the ventral tegmental area (VTA) in the state-dependent memory

The VTA is a structure of the midbrain and contains the cell bodies of the mesolimbic dopaminergic system, which projects the dopaminergic pathways to several corticolimbic structures, such as the hippocampus. Activation of the VTA causes dopamine release in the corticolimbic structures (for example the hippocampus) and seems to have a key role in hippocampal plasticity (Nazari-Serenjeh et al., 2011[43]). Moreover, the interaction between diverse mechanisms, e.g., opioidergic, cholinergic, and glutamatergic in the VTA may affect learned behaviors (Ahmadi et al., 2007[1]). For example, the activation and blockage of VTA mAChRs and nAChRs may modify morphine state-dependent learning (Darbandi et al., 2008[13]; Rezayof et al., 2008[60]). Some experiments reported that bilateral infusion of atropine within the VTA inhibited morphine state-dependent learning in the passive avoidance learning test (Darbandi et al., 2008[13]). Moreover, other investigations exhibited that the application of nicotine into the VTA improved morphine state-dependent learning in the passive avoidance test (Rezayof et al., 2008[60]). As mentioned above, morphine state-dependent learning appears to be a kind of reward-associated learning. Moreover, the role of VTA cholinergic receptors in reward-related learning confirmed by a study in which microinjection of cholinergic agents in the VTA improved morphine-produced place preference. Therefore, it is probable that co-injection of cholinergic drugs with morphine improved the reward effects and produced state-dependent learning (Darbandi et al., 2008[13]; Rezayof et al., 2008[60]). Additionally, the influence of nicotine on the retrieval of morphine state-dependent learning may be due to the NMDA receptors of VTA. Nicotine and NMDA may participate with morphine on various signaling properties that are necessary for the state under which learning occurs (Ahmadi et al., 2007[1]).

### Involvement of the nucleus accumbens (NAC) in the state-dependent memory

**Figure 5:**
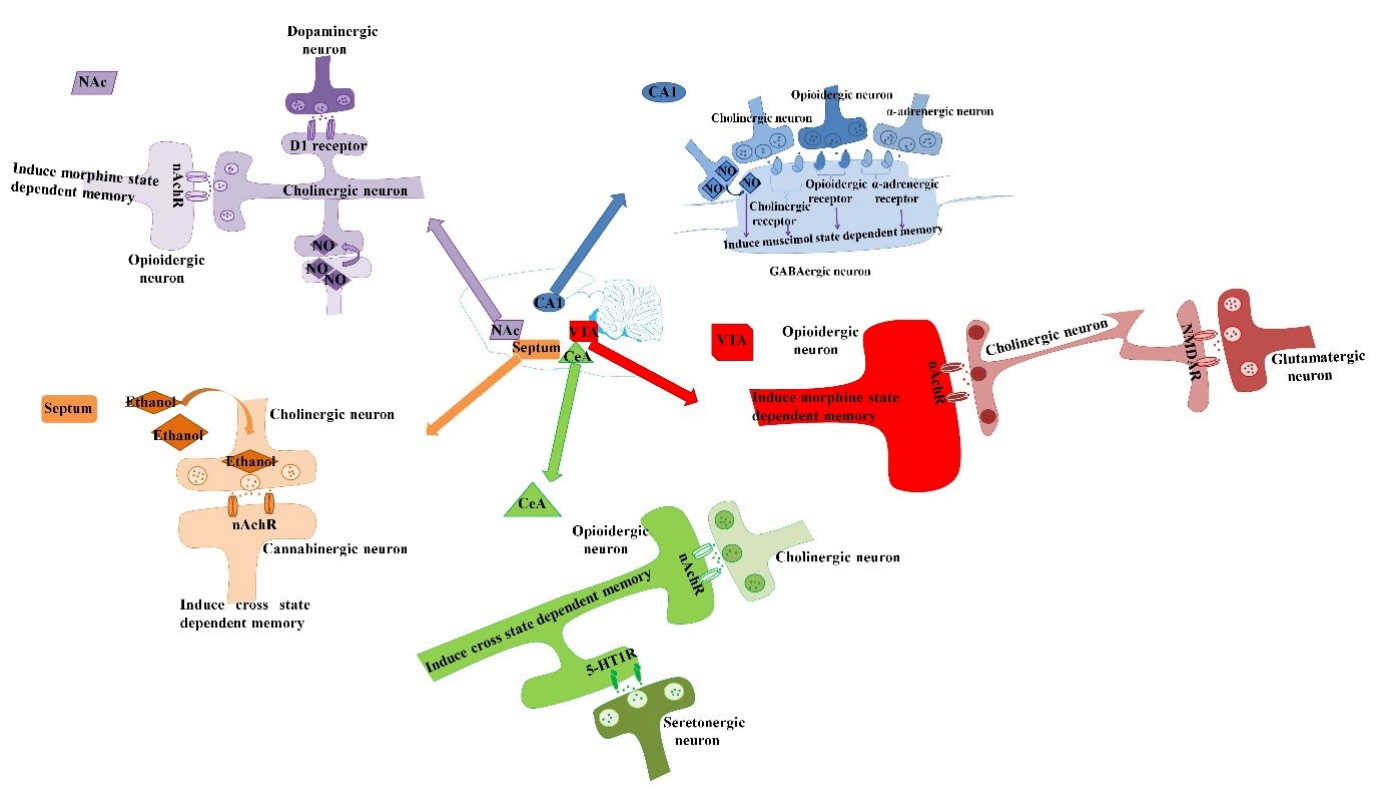
Schematic illustration of the effect of different brain areas and various neurotransmitter mechanisms in the modulation of state-dependent memory induced by morphine. Some documents reported that opioidergic (Jafari-Sabet and Jannat-Dastjerdi, 2009), muscarinic cholinergic (Jafari-Sabet, 2011), and α2-adrenergic (Jafari-Sabet et al., 2013), systems in the CA1 area are essential for inducing muscimol-related state-dependent memory (Jafari-Sabet et al., 2014). Moreover, cholinergic and serotonergic receptor systems of the CeA play a key role in morphine-induced state-dependent memory (Tirgar et al., 2014). In the medial septum, there is cross state-dependent memory retrieval between cannabinoid and acetylcholine or ethanol (Alijanpour and Rezayof, 2013). Also, cholinergic and glutamatergic receptors of the VTA participate in morphine state-dependent learning (Ahmadi et al., 2007). Also, nitric oxide, cholinergic and dopaminergic systems, of the NAc are involved in morphine state-dependent learning (Zarrindast et al., 2012b). CeA: central nucleus of the amygdala, VTA: ventral tegmental area, and NAc: nucleus accumbens

The NAC is an important site of the ventral striatum which is well known for its function in modulating the reward, learning, movement, attention, and motivation. It has been revealed that NAC and VTA have reciprocal connections. Also, there is a functional correlation among the midbrain dopaminergic neurons and the hippocampus, whose stimulation causes the improvement of LTP and learning (Zarrindast et al., 2012[89]). The NAC involved in passive avoidance learning and morphine state-dependent learning, too (Noorbakhshnia and Zarrinimehr, 2019[48]). Reports are suggesting a probable role for the dopaminergic (Azizbeigi et al., 2011[5]), and NO (Zarrindast et al., 2012[89]) systems of the NAC in the improving role of nicotine on the morphine-produced memory-impairment as well as morphine state-dependent memory (Zarrindast et al., 2012[89]). Investigations revealed that abuse of drugs, for instance, morphine and nicotine which are administrated systemically by addicts induce their properties via functions in the limbic component of the basal ganglia, especially the NAC. In animals, micro-dialysis investigations have displayed that systemic injection of such addictive drugs specially enhanced the extracellular DA level in the NAC. The document demonstrated that morphine and nicotine induce their rewarding properties through stimulation of DA release in the NAC. Moreover, nicotine by stimulation of the DA receptors in the NAC may increase retrieval of memory-impaired by morphine (Azizbeigi et al., 2011[5]). It has been revealed that nicotine-caused recovery of morphine amnesia increased or decreased via inhibition or stimulation of the NAC NO system, respectively (Figure 5(Fig. 5); References in Figure 5: Ahmadi et al., 2007[1]; Alijanpour and Rezayof, 2013[2]; Jafari-Sabet and Jannat-Dastjerdi, 2009[28]; Jafari-Sabet et al., 2013[27], 2014[30]; Jafari-Sabet, 2011[26]; Tirgar et al., 2014[73]; Zarrindast et al., 2012[89]) (Zarrindast et al., 2012[89]).

## Conclusion

Studies have shown that state-dependent learning is produced via various exogenous and endogenous compounds, whereby exogenous state-dependent learning is induced by addictive drugs and endogenous state-dependent learning is produced via neuro-humoral states. Several reports demonstrated that many neurotransmitters are involved in state-dependent learning. Additionally, many regions of the brain, including the CA1 areas of the hippocampus, amygdala (especially CeA), septum, VTA, and NAc, participated in state-dependent learning and memory. Interestingly, there are cross state-dependent learning and memory among some drugs that may well prove a novel natural treatment for memory deficiency disease. According to this researches, we proposed that different brain areas due to having many neurotransmitter systems and many afferent and efferent projections play the main role in the modulation of state-dependent memory. Moreover, we suggested that the administration of some drugs (systemically or intra-cerebrally) impaired memory but re-administration of the drugs on the same state could improve memory (inducing state-dependent memory) which depends on the time-, dose- and half-life of drugs (Nishimura et al., 1990[47]; Khavandgar et al., 2003[35]; Osorio-Gomez et al., 2019[50]).

## Acknowledgements

We are thankful to all contributors for their participation. Also, the authors would like to express their appreciation to Dr. Mina Hajifaraj Tabrizi and Dr. Zhaleh Moazzeni for editing the manuscript. 

## Authors’ contributions

Khakpai F wrote the manuscript. Zarrindast MR was responsible for the study concept, design, and interpretation of findings. The authors critically reviewed the content and approved the final version for publication.

## Conflict of interest

There is no conflict of interest in this manuscript.

## Figures and Tables

**Table 1:**
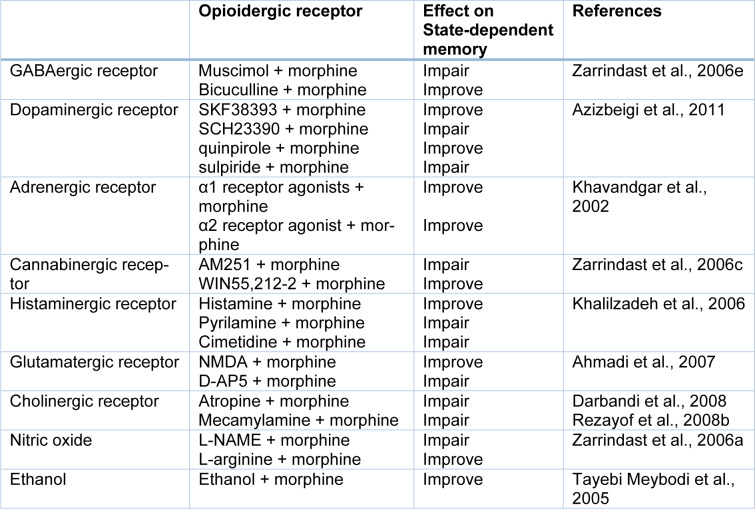
Effect of various neurotransmitters and pharmacological compounds on state-dependent memory produced by morphine

**Table 2:**
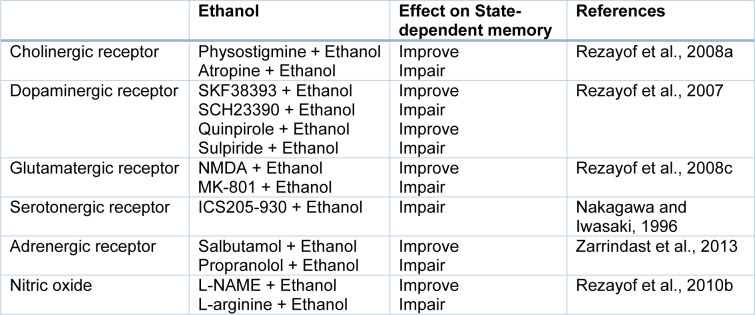
Effect of some neurotransmitters on state-dependent memory elicited by ethanol

**Table 3:**
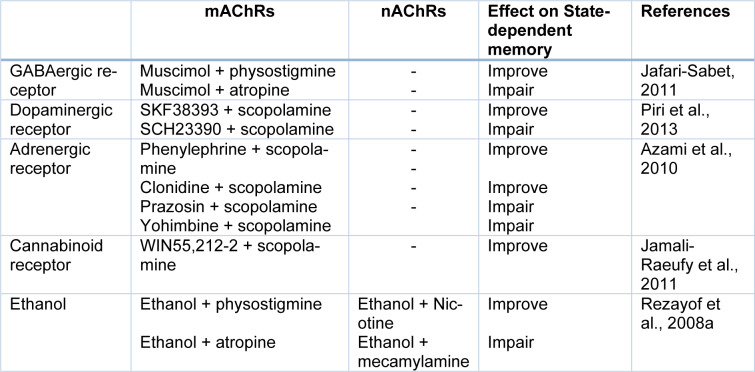
Effect of some neurotransmitters and pharmacological compounds on state-dependent memory induced by acetylcholine

**Figure 1:**
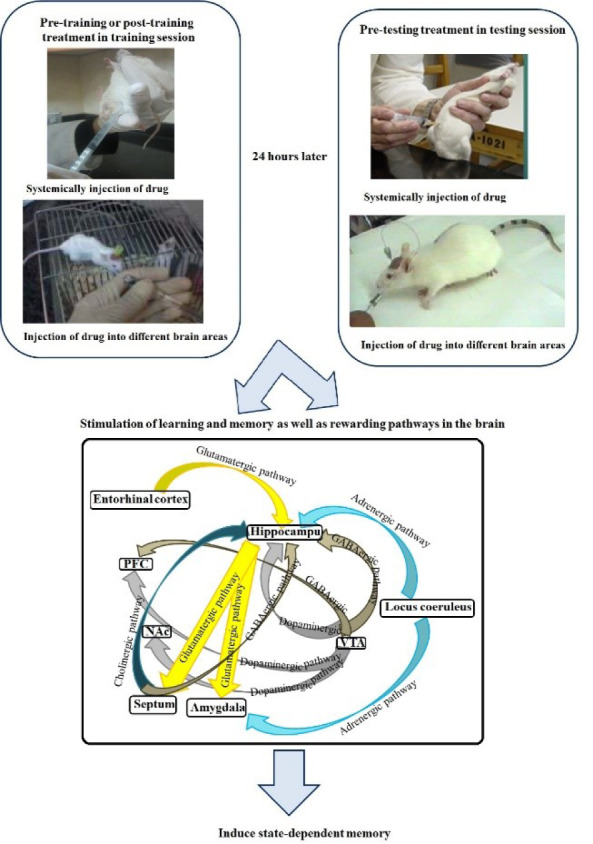
Graphical abstract
